# Stages of HPV Vaccine Hesitancy Among Guardians of Female Secondary School Students in China

**DOI:** 10.1016/j.jadohealth.2022.08.027

**Published:** 2023-01

**Authors:** Zheng Wei, Yang Liu, Liuren Zhang, Xiu Sun, Qijing Jiang, Zhenwei Li, Yue Wu, Chuanxi Fu

**Affiliations:** a Institute of Infectious Disease and Vaccine, School of Public Health, Zhejiang Chinese Medical University, Hangzhou, China; b Department of Infectious Disease Epidemiology, Faculty of Epidemiology and Population Health, London School of Hygiene and Tropical Medicine, Bloomsbury, London, England, UK; c Centre for Mathematical Modelling of Infectious Diseases, London School of Hygiene and Tropical Medicine, Bloomsbury, London, England, UK

**Keywords:** HPV vaccine, Vaccine hesitancy, Adolescent health

## Abstract

**Purpose:**

Female secondary school students are the primary recommended population for human papillomavirus (HPV) vaccines. However, vaccine hesitancy may affect uptake. In this study, we assessed the vaccine hesitancy levels among the guardians of female secondary school students in China.

**Methods:**

We developed a questionnaire and conducted cross-sectional surveys among guardians of secondary school girls aged 12–19 years in mainland China based on the Increasing Vaccination Model and the Precaution Adoption Process Model.

**Results:**

We collected 3,225 valid samples. Among the participating guardians, 53.9% were vaccine hesitant, although only 0.9% had refused HPV vaccines. Some individual characteristics of guardians (e.g. sex, education/income level) were associated with understanding HPV vaccines. Better knowledge of HPV vaccines and communication with reliable sources of information were associated with vaccine nonhesitancy. Practical barriers such as vaccine shortage and busy schedules prevented nonhesitant guardians from vaccinating their children.

**Discussion:**

A substantial proportion of the guardians surveyed were HPV vaccine hesitant. Promoting HPV knowledge and communication with reliable sources (e.g. clinical doctors) could help fight against vaccine hesitancy.

**Implications and Contribution**

HPV vaccine hesitancy is common among guardians of secondary school girls in mainland China. Relevant knowledge promotion campaigns, particularly through clinical doctors, may improve vaccine uptake. Vaccine non-hesitant guardians may fail to vaccinate their children due to logistic challenges. Resources should be committed to addressing these challenges.

Vaccines are considered one of the most effective public health interventions, saving millions of lives each year [1]. Nevertheless, vaccine hesitancy may hinder the global efforts against vaccine preventable diseases. The World Health Organization (WHO) has now recognized vaccine hesitancy as one of the biggest threats to global health [2,3]. The recent measles outbreaks [4,5], the low influenza vaccine coverage [6,7], and the public distrust in the COVID-19 vaccine may be associated with vaccine hesitancy [3,8].

Persistent infection by oncogenic human papillomavirus (HPV) subtypes precedes the development of cervical cancer, which affects 528,000 women annually, causing 266,000 deaths worldwide [9]. In China, there are approximately 106,000 new cervical cancer cases and 48,000 related deaths each year [10]. The number of cervical cancer diagnoses will likely continue to increase due to the aging population [11]. HPV vaccines have been proven to prevent vaccine-type cervical cancer effectively. The WHO calls for actions toward eliminating cervical cancer and recommends girls aged 9–14 years as the primary target population for HPV vaccination [12]. However, vaccine hesitancy strongly affects the uptake of this vaccine [13,14].

By March 31, 2017, 71 countries had introduced HPV vaccines into national immunization programs (NIPs) for girls only and 11 countries for both girls and boys [12]. In China, however, HPV vaccination is not yet a part of the NIP and relies on out-of-pocket payments by the guardians. Consequently, cervical cancer burden has remained high while the HPV vaccine coverage remained low [15]. The high price, the supply shortage, and the lack of awareness of the HPV vaccine may have affected HPV vaccine uptake [16]. Moreover, the recent vaccine safety incidents have inevitably undermined public trust in vaccines [[17], [18], [19]]. However, the magnitude of impacts due to these factors have yet to be quantified using evidence in mainland China.

This study validated a survey tool and assessed the magnitude of HPV vaccine hesitancy among guardians (i.e. parents, in this study) of female secondary school students (aged 12–19 years) in mainland China using cross-sectional surveys. The exact definition of vaccine hesitancy may vary. Initially, vaccine hesitancy was defined as an intermediate attitude between accepting and rejecting vaccinations [20,21]. In 2012, WHO’s Strategic Advisory Group of Experts on Immunization (SAGE) defined vaccine hesitancy as the behavior of delaying or refusing vaccines despite vaccine availability that hinders the success of immunization programs [21,22]. The distinction between attitude and behavior [6,23], while studying vaccine hesitancy, has made it difficult to compare and synthesize results. In this study, we applied the Increasing Vaccination Model (IVM) recently proposed by SAGE, combining attitude and behavior elements [24]. This study also identifies practical barriers in vaccination programs, generating evidence that may inform future intervention planning.

## Methods

**Figure 1:**
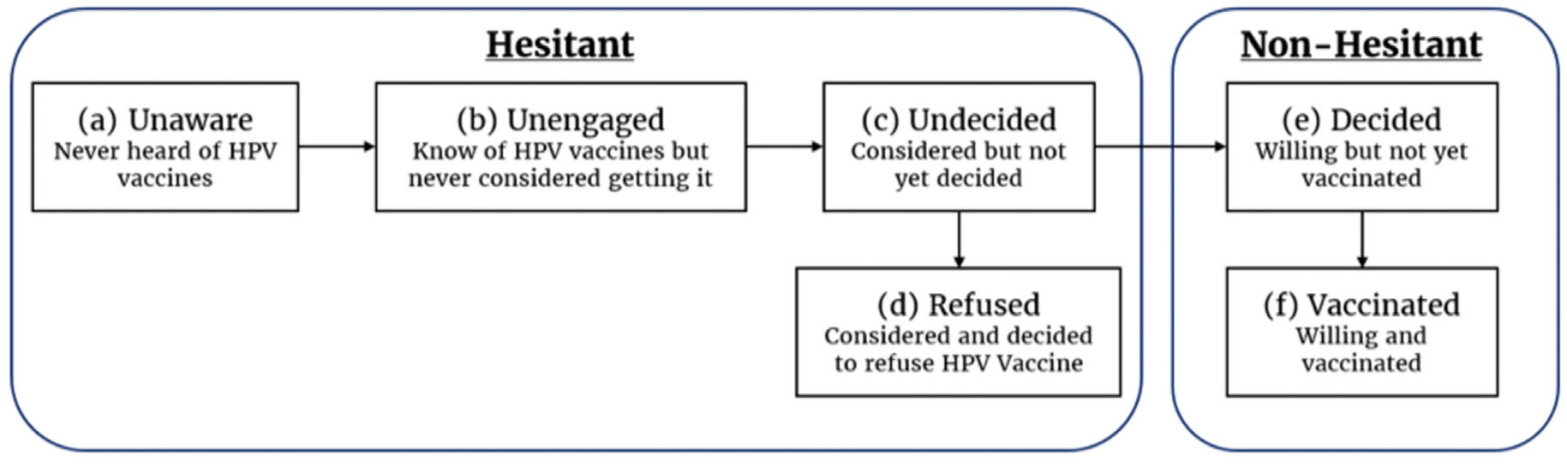
Stages of vaccine attitudes and behaviors based on the Increasing Vaccination Model and the Precaution Adoption Process Model. Stages (a–d) are hesitant stages and (e-f) nonhesitant stages.

We used two approaches to understanding vaccine hesitancy. Under the framework of the IVM proposed by WHO's SAGE in 2017, perceptions (e.g. safety concerns) and social processes (e.g. information sharing) determine the motivation to vaccinate, while practical issues (e.g. costs) determine the vaccination outcomes [24]. Under the Precaution Adoption Process Model framework, the decision-making process is continuous and dynamic, ranging from complete unawareness to action [25]. Building on these two models, we categorized the decision-making process related to HPV vaccination into six stages (Figure 1).

### Questionnaires and participants

We developed a six-part questionnaire with 46 questions (Table 1) to investigate the association between individual characteristics and vaccine attitudes and behavior stages (see Supplemental Methods for complete questionnaire). This questionnaire additionally included quality control components to detect inattentive or unmotivated participants. The HPV vaccine hesitancy scale in this study (item 3 in Table 1) was created mainly based on the Vaccine Hesitancy Scale developed by the WHO SAGE Working Group on Vaccine Hesitancy generally for all vaccines [25]. We modified this scale for studying HPV vaccine hesitancy based on existing research [[26], [27], [28], [29], [30]], the Delphi technique, and a pilot survey. The results in this study are presented using a five-point Likert scale. We used a cutoff value of 3—a score lower than three indicates vaccine hesitancy [31,32].

**Table 1:** Structure of questionnaire

Component index	Broad category	Brief description/examples
1	Socioeconomic and demographic factors	Sex and education level of guardian, age of guardian and student, annual household income
2	Vaccine hesitancy status	Six stages of vaccine hesitancy
2+ (conditional extension)	HPV vaccine–related knowledge	E.g. Hesitant guardians were asked about their decisions if the HPV vaccines were free; nonhesitant guardians were asked about their preferences across different HPV vaccines (i.e. 2, 4, and 9 valences).
3	HPV vaccine hesitancy scale^a^	Personal perceptions toward HPV vaccination (necessity, safety, importance)
4	HPV vaccine conspiracy beliefs scale^b^	Trust of guardians toward pharmaceutical companies
5	Social processes	The extent to which the guardians' attitudes are affected by those around them
6	Practical barriers	E.g. Awareness of points of vaccination and price perceptions; Only applicable to nonhesitant guardians

a These items were developed based on existing literature for this study [[21], [22], [23], [24], [25]]. There were two rounds of Delphi consultation and pilot survey, based on which the items were revised for improved validity, rationality, and readability.

b Five-point Likert scale ranges from “strongly disagree” to “strongly agree.” Variables were reverse coded before analysis—a high score on the HPV vaccine conspiracy beliefs scale indicates negative perceptions, and a low score indicates positive perceptions.

We used the conspiracy beliefs scale (item 4 in Table 1) to understand how vaccine conspiracy beliefs affect vaccine hesitancy and uptake [33]. Initially developed in 2016, this tool has been widely used and validated. This scale also uses a five-point system. A high score indicates negative vaccine perceptions as a result of reverse coding before data analysis.

Between May and June 2020, we conducted surveys on HPV vaccine hesitancy via the Wenjuanxing online platform [34]. We limited participants to guardians (defined as the parents, in this study) of 12–19 years old secondary school girls. Participants were solicited using two mechanisms. First, with the help of the Centers for Disease Control and Prevention in Hangzhou (capital of Zhejiang province in eastern China) and Guangzhou (capital of Guangdong province in southern China), we solicited participants from two middle schools (12–15 years) and two high schools (16–19 years) by having the homeroom teachers shared the link to the online questionnaire with all guardians of female students. Their questionnaires are hereinafter referred to as “field samples.” Second, we solicited participants among Wenjuanxing platform’s users nationwide. Wenjuanxing promoted this questionnaire through social media as a paid opportunity—guardians who finished the questionnaire received a monetary incentive. Their questionnaires are hereinafter referred to as the “online samples.”

### Statistical analyses

We conducted exploratory factor analysis (EFA) on the field samples using principal axis factoring with orthogonal rotation to explore the relational structure of the vaccine hesitancy or conspiracy beliefs scales [25]. Cronbach’s α was calculated to determine the reliability (i.e. internal consistency of latent variables identified via EFA, hereafter “dimension”) of the measurement scales used. We then conducted confirmatory factor analysis on the online sample to examine the model (identified via EFA) fit. Independent *t* test, analysis of variance, and post hoc *t* test were used to evaluate the differences in scale scores by vaccine hesitancy stages and individual characteristics. Chi-square tests were used to analyze the impacts of HPV vaccine related knowledge, practical barriers, and social factors on HPV vaccination attitude and behavior.

Statistical analyses were performed using SPSS version 25.0 0 (IBM Corporation, New York, NY) and Amos 21.0. Results visualisation relied on R (v.4.1.0).

The ethics committee of Zhejiang Chinese Medical University reviewed and approved this protocol (#B20191205). We obtained informed consent from all survey participants.

## Results

### Descriptive statistics and model validation

We collected 868 field samples (512 in Hangzhou and 356 in Guangzhou) and 4,927 online samples; 169 field and 2,401 online samples were subsequently deleted using the quality control components. The results we presented below were based on the remaining 3,225 samples. Among the guardians surveyed, 65.2% had middle school–aged daughters, 71.3% were mothers, 72.1% lived in urban areas, 76.2% had a bachelor’s degree or above, and 55.2% had an annual household income above US$15,000 (i.e. the average household income of China in 2020) [35].

With our results on the HPV Vaccine Hesitancy Scale, the Kaiser-Meyer-Olkin measure of sampling adequacy was 0.836; Bartlett’s test of sphericity returned *p* values smaller than .001, both indicating that factor analysis may be useful.

The EFA (n = 699, field samples only) identified three dimensions with eigenvalues greater than 1, which explained 63.82% of the common variance among 14 items. The item “Young girls vaccinated against HPV may be perceived by others to be more sexually active” was deleted as the standardized factor loading was smaller than 0.6, which indicated that it does not contribute to the dimensional construct identified through the EFA. The remaining 14 items had factor loading above 0.6 and no cross loading above 0.4.

Based on the items that fell under each dimension, we named these dimensions identified as “necessity,” “importance,” and “safety”. The “necessity” dimension included items such as “my daughter is in good health and does not need to be vaccinated against HPV.” The “importance” dimension included items such as “HPV vaccination is very important to my daughter’s health.” The “safety” dimension included items such as “I am worried about my daughter having side effects from HPV vaccines.” A complete list of item-and-dimension pairs can be found in Supplemental Table 1.

For the HPV vaccine conspiracy beliefs scale, the EFA identified only one item with eigenvalues greater than 1, which explained 76.80% of the common variance of all eight items. Cronbach’s α for dimensions “necessity,” “importance,” “safety,” and HPV vaccine conspiracy beliefs scale were 0.876, 0.821, 0.786, and 0.957—indicating scale reliability. The confirmatory factor analysis (n = 2,526, online samples only) results indicated a valid factor structure. Detailed factor analysis and model validation results can be found in Supplemental Tables 1–3

### Stage distribution among guardians

Among 3,225 guardians who participated in the survey, 53.9% experienced vaccine hesitancy, but only 0.9% had refused HPV vaccines. The unaware, unengaged, and undecided stages accounted for 14.2%, 17.7%, and 21.1% of the total participants. Although 46.1% of guardians were vaccine nonhesitant, only the daughters of 7.5% were vaccinated for HPV.

**Figure 2:**
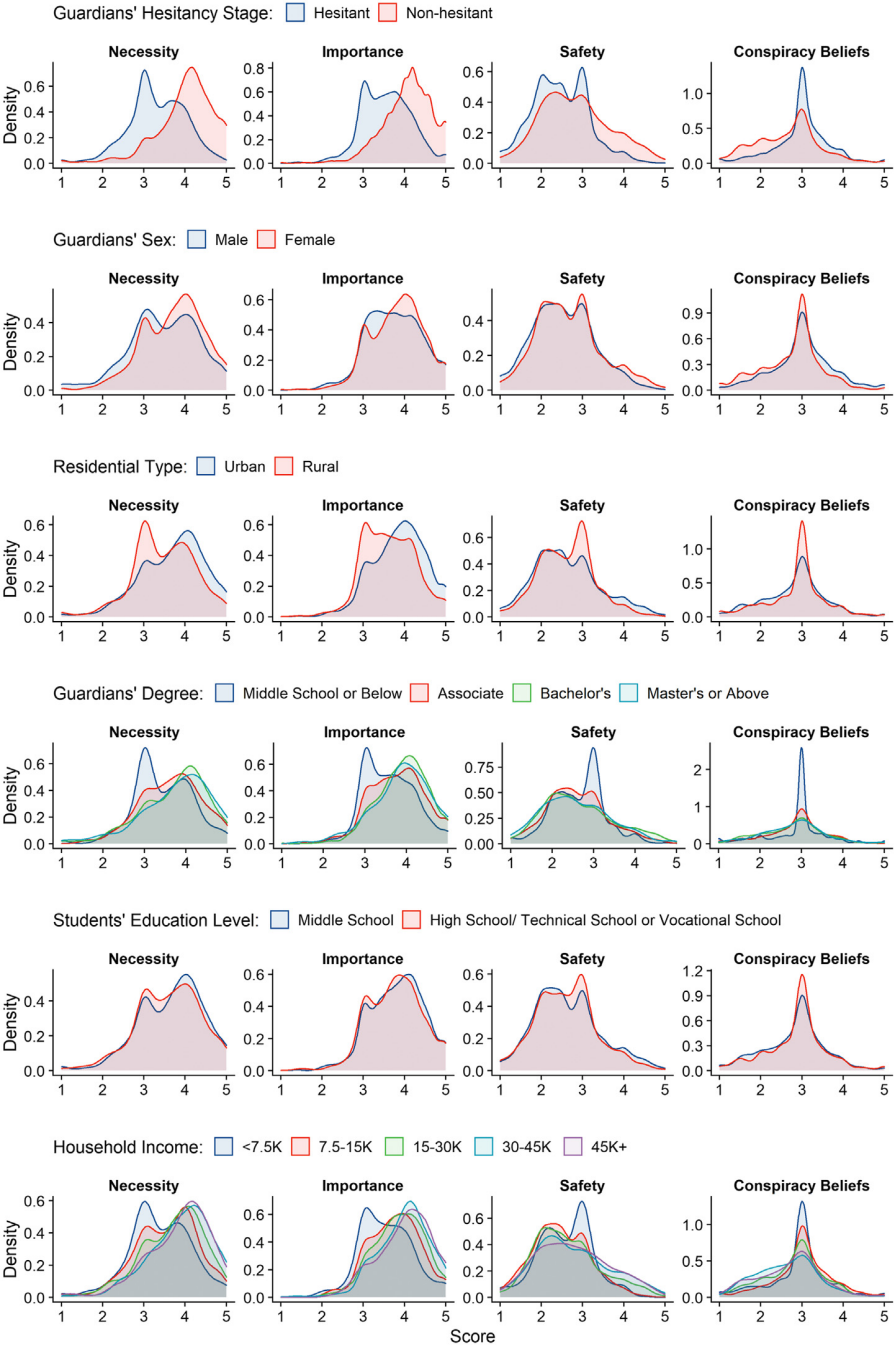
Probability density functions of scores in vaccine necessity, importance, safety, and HPV vaccine conspiracy beliefs and individual characteristics of participating guardians and their children.

We found associations between vaccine hesitancy and individual characteristics (Figure 2). Vaccine nonhesitant or female guardians scored higher in “necessary,” “importance,” and “safety” dimensions and lower in terms of conspiracy beliefs (*p* < .01). Guardians who resided in urban areas or had bachelor’s degrees or above scored higher in the “necessity” and “importance” dimensions (*p* < .001). The “safety” dimension and HPV vaccine conspiracy beliefs were not associated with guardian residential status (urban or rural) or education level (*p* > .05).

Annual household income was associated with higher scores in the “necessity” and “importance” dimensions (*p* < .01). Only very high annual household income levels (i.e. 30K+) are associated with higher scores in the “safety” dimension and lower scores on the HPV vaccine conspiracy beliefs scales (*p* < .01). Compared to guardians of students aged 16–19, those of students aged 12–15 scored higher in the “necessity” dimension and the HPV vaccine conspiracy beliefs scale (*p* < .05)—there are no significant differences between the two groups in the “importance” and “safety” dimensions (*p* > .05). The itemizs`d score by metric and the related test statistics can be found in Supplemental Table 4.

### Knowledge, barriers, and social factors

**Figure 3:**
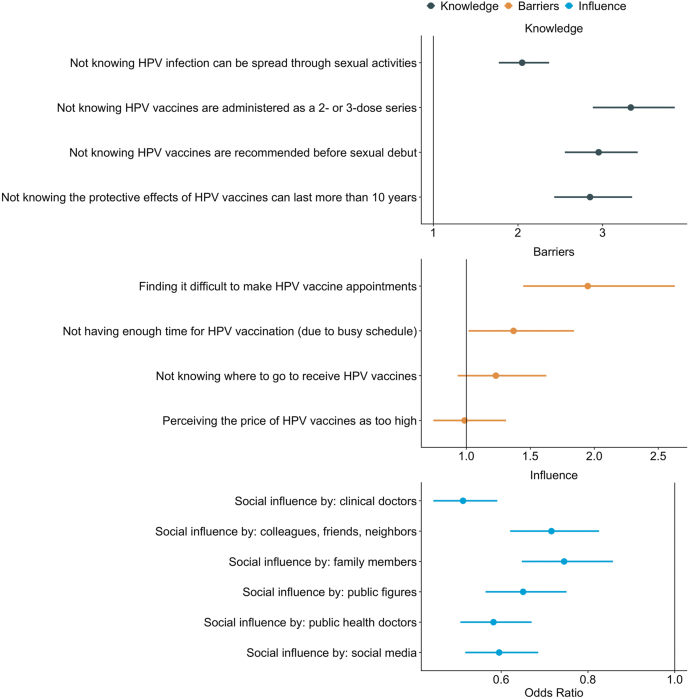
The associations between vaccine hesitancy versus knowledge on HPV and social influences (with guardians as influence recipients) and the association between the transition between “decided” and “vaccinated” stages and practical barriers. The specific values used to create these figures can be found in the Appendix.

We calculated the odds ratio to identify the factors associated with HPV vaccine hesitancy among guardians of female secondary school students in China (Figure 3). For example, the odds of vaccine hesitancy among guardians who did not know that HPV infection can be spread via sexual activities was 2.05 (95% confidence interval (CI), 1.77–2.37) times the odds of vaccine hesitancy among guardians who do know. Overall, we showed that a lack of knowledge on HPV infections and vaccines is associated with vaccine hesitancy.

We examined the barriers that stopped people from transitioning from “decided” to “vaccinated” using the same approach. For example, the odds of having decided but not having had their children vaccinated among guardians who found it difficult to get an appointment for HPV vaccination is 1.95 (95% CI, 1.44–2.63) times the odds of those who have decided and vaccinated their children. Besides the difficulty in booking an appointment, not having time in their busy schedule also explained why some “decided” had not yet vaccinated their daughters. The perception of the HPV vaccine prices and the knowledge of available points of vaccinations were not associated with vaccine hesitancy based on our results.

All sources of social influences we investigated were associated with nonhesitancy among guardians. The strongest associations were found for clinical doctors. The odds of vaccine hesitancy among guardians who said their perspectives were influenced by clinical doctors were 0.51 (95% CI, 0.44–0.59) times the odds of vaccine hesitancy among guardians who claimed otherwise.

The results behind Figure 3 are also presented in Supplemental Tables 5–7.

### Further insights into hesitant and nonhesitant guardians

We asked the preferences of specific vaccine products among guardians in the “decided” and “vaccinated” stages and found that generally, guardians preferred higher valent products. However, approximately one-third of guardians in these stages did not know the implication of differences in vaccine valence covered.

We asked hesitant guardians if they would like to have their children vaccinated if HPV vaccines were introduced into the NIP (through which they would be free). Most guardians in hesitant stages said they would then vaccinate their children. The most drastic changes were observed among “unengaged” and “undecided” guardians, while the smallest changes were observed among “refused” guardians. More details on the results regarding the vaccine product preferences and the impact of central government funding can be found in Supplemental Table 8.

## Discussion

We conducted a cross-sectional survey in mainland China to understand HPV vaccine hesitancy among guardians of school girls aged 12–19 years. Our approach combined IVM and Precaution Adoption Process Model. We developed and validated a reliable scale for measuring the HPV vaccine hesitancy among guardians.

Among our survey participants, 54% were vaccine hesitant, comparable to 58% found in the United States and 62% in France [36,37]. Although the proportion of HPV vaccine refusal was low (0.9%), nearly one-third of the guardians had never heard of or considered vaccinating their children against HPV, which may have contributed to the low vaccine coverage observed in mainland China [16]. Similar phenomena have been found in Romania, the Netherlands, and Denmark [38]. Overall, female guardians, guardians who reside in urban settings, guardians with higher levels of education, and guardians with a higher annual household income had a better understanding of the necessity, importance, and safety of the HPV vaccines and were influenced less by HPV vaccine conspiracy beliefs.

Safety was a common reason for guardians to decide against HPV vaccination in this study, consistent with existing literature [39,40]. In the WHO/UNICEF Joint Report Form on vaccine hesitancy, safety concerns are also the main contributor to global vaccine hesitancy [41]. Substantial declines in HPV vaccine uptake due to safety concerns have been observed [36]. Between 2003 and 2018, 39 vaccine safety–related events were reported in mainland China, leading to negative media coverage that has seriously undermined the public confidence in vaccines [17,[42], [43], [44]].

We showed that guardians’ knowledge of HPV and HPV vaccines was poor. Consistently with existing evidence, we highlighted that this lack of knowledge was associated with HPV vaccine hesitancy [45]. Social influences were associated with HPV vaccine nonhesitancy. Communicating with clinical doctors may be a particularly effective pathway to promoting the benefits of HPV vaccines. Existing research suggested that social benefit programs, communication with health-care workers, and print media (e.g. newspapers/magazines) may also help promote HPV and HPV vaccine-related knowledge in China [46].

In this study, although 46.1% of guardians were vaccine nonhesitant, only 7.5% vaccinated their children. The barriers to implementation include challenges in booking an appointment (due to vaccine shortage) and finding time in busy schedules. Our study found that perceptions on price were not associated with HPV vaccine hesitancy, inconsistent with some existing evidence [47].

It was worth noting that female secondary school students may not be able to complete the entire schedule after the initial dose [9]. The proportion of female secondary school students who have completed their HPV vaccine schedule may be even lower than 7.5%. More robust logistical support (e.g. secure vaccine supply, school-based vaccination programs) may be required to boost HPV vaccine coverage in the future.

Introducing the HPV vaccines into NIP in mainland China, making it free for all, could incentivize more guardians to vaccinate their children [48]. The introduction may be facilitated by the recently authorized domestically produced bivalent HPV vaccine (against strains 16 and 18), Cecolin (Innovax, Xiamen, China). The price of Cecolin is half that of Cervarix (GlaxoSmithKline, also bivalent and against strains 16 and 18) while showing similar efficacy [15]. However, further health education and communication might be needed as we found that a substantial proportion of nonhesitant guardians did not know the differences between vaccine products [43].

This study has several limitations. First, the cross-sectional study design allowed us to identify associations but not causality. Second, the study pooled online and field samples via a web-based platform. Both sets of samples may involve biases due to factors such as accessibility (e.g. we could not reach those with no access to the Wenjuanxing platform at all) or monetary incentives (only applicable to online samples). As a result, the guardians sampled were disproportionally female, urban, and well educated. Third, we did not collect information on geographic locations where participants’ children reside among online samples. Different regions of China may not have been equally represented. Lastly, the instrument developed in this study could benefit from further validation using different data sets.

### Conclusions

In this study, we assessed the magnitude of vaccine hesitancy among guardians of female secondary school students aged 12–19 years in mainland China using a cross-sectional survey. Among survey participants, 54% were vaccine hesitant and 32% were unaware of HPV vaccines or had never considered vaccinating their children against HPV. Guardians’ individual characteristics (e.g. sex, income, and education level) are associated with their perceptions of the necessity, importance, and safety of the HPV vaccines and how strongly they are affected by vaccine conspiracy beliefs. Knowledge of HPV vaccines and social influences (e.g. communication with clinical doctors) could help fight against vaccine hesitancy. Vaccine shortage and busy schedules were the main barriers preventing implementation among the guardians who have already decided to vaccinate their children.

## References

[1] World Health Organization Vaccines and immunisation. https://www.who.int/health-topics/vaccines-and-immunization.

[2] World Health Organization (2019). Ten threats to global health. https://www.who.int/emergencies/ten-threats-to-global-health-in-2019.

[3] The Lancet Child Adolescent Health (2019). Vaccine hesitancy: A generation at risk. Lancet Child Adolesc Health.

[4] Lo N.C., Hotez P.J. (2017). Public health and economic consequences of vaccine hesitancy for measles in the United States. JAMA Pediatr.

[5] Paules C.I., Marston H.D., Fauci A.S. (2019). Measles in 2019 - going backward. N Engl J Med.

[6] Schmid P., Rauber D., Betsch C. (2017). Barriers of influenza vaccination Intention and behavior - a systematic review of influenza vaccine hesitancy, 2005 - 2016. PLoS One.

[7] Larson H.J. (2018). The state of vaccine confidence. The Lancet.

[8] Lin C., Tu P., Beitsch L.M. (2020). Confidence and receptivity for COVID-19 vaccines: A rapid systematic review. Vaccines (Basel).

[9] Arbyn M., Weiderpass E., Bruni L. (2020). Estimates of incidence and mortality of cervical cancer in 2018: A worldwide analysis. Lancet Glob Health.

[10] World Health Organization (2017). Human papillomavirus vaccines: WHO position paper, May 2017-Recommendations. Vaccine.

[11] Malagón T. (2019). Reasons for optimism about eliminating cervical cancer in China. Lancet Public Health.

[12] World Health Organization (2017). Summary of the WHO position paper on vaccines against human papillomavirus (HPV). https://www.who.int/immunization/position_papers/pp_hpv_oct2014_summary.pdf.

[13] Szilagyi P.G., Albertin C.S., Gurfinkel D. (2020). Prevalence and characteristics of HPV vaccine hesitancy among parents of adolescents across the US. Vaccine.

[14] Sweileh W.M. (2020). Bibliometric analysis of global scientific literature on vaccine hesitancy in peer-reviewed journals (1990-2019). BMC Public Health.

[15] Zou Z., Fairley C.K., Ong J.J. (2020). Domestic HPV vaccine price and economic returns for cervical cancer prevention in China: A cost-effectiveness analysis. Lancet Glob Health.

[16] Zhao F., Qiao Y. (2019). Cervical cancer prevention in China: A key to cancer control. Lancet.

[17] Yang R., Penders B., Horstman K. (2019). Addressing vaccine hesitancy in China: A Scoping review of Chinese Scholarship. Vaccines (Basel).

[18] Yu W., Cao L., Liu Y. (2020). Two media-reported vaccine events in China from 2013 to 2016: Impact on confidence and vaccine utilization. Vaccine.

[19] Simms K.T., Hanley S.J.B., Smith M.A. (2020). Impact of HPV vaccine hesitancy on cervical cancer in Japan: A modelling study. Lancet Public Health.

[20] Larson H.J., Jarrett C., Eckersberger E. (2014). Understanding vaccine hesitancy around vaccines and vaccination from a global perspective: A systematic review of published literature, 2007-2012. Vaccine.

[21] World Health Organization Report of the SAGE working group on vaccine hesitancy. http://www.who.int/immunization/sage/meetings/2014/october/.

[22] MacDonald N.E., Hesitancy SWGoV. (2015). Vaccine hesitancy: Definition, scope and determinants. Vaccine.

[23] Quinn S.C., Jamison A.M., An J. (2019). Measuring vaccine hesitancy, confidence, trust and flu vaccine uptake: Results of a national survey of White and African American adults. Vaccine.

[24] Brewer N.T., Chapman G.B., Rothman A.J. (2017). Increasing vaccination: Putting psychological science into action. Psychol Sci Public Interest.

[25] Shapiro G.K., Tatar O., Dube E. (2018). The vaccine hesitancy scale: Psychometric properties and validation. Vaccine.

[26] Perez S., Shapiro G.K., Tatar O. (2016). Development and validation of the human papillomavirus attitudes and beliefs scale in a national Canadian sample. Sex Transm Dis.

[27] Betsch C., Schmid P., Heinemeier D. (2018). Beyond confidence: Development of a measure assessing the 5C psychological antecedents of vaccination. PLoS One.

[28] Dudley M.Z., Privor-Dumm L., Dubé È., MacDonald N.E. (2020). Words matter: Vaccine hesitancy, vaccine demand, vaccine confidence, herd immunity and mandatory vaccination. Vaccine.

[29] Shapiro G.K., Tatar O., Amsel R. (2018). Using an integrated conceptual framework to investigate parents’ HPV vaccine decision for their daughters and sons. Prev Med.

[30] Perez S., Tatar O., Gilca V. (2017). Untangling the psychosocial predictors of HPV vaccination decision-making among parents of boys. Vaccine.

[31] Luyten J., Bruyneel L., van Hoek A.J. (2019). Assessing vaccine hesitancy in the UK population using a generalized vaccine hesitancy survey instrument. Vaccine.

[32] Kempe A., Saville A.W., Albertin C. (2020). Parental hesitancy about Routine childhood and influenza vaccinations: A national survey. Pediatrics.

[33] Shapiro G.K., Holding A., Perez S. (2016). Validation of the vaccine conspiracy beliefs scale. Papillomavirus Res.

[34] Ning L., Niu J., Bi X. (2020). The impacts of knowledge, risk perception, emotion and information on citizens’ protective behaviors during the outbreak of COVID-19: A cross-sectional study in China. BMC Public Health.

[35] National Bureau of Statistics National data-data query: Average household income of China in 2020. http://www.stats.gov.cn/tjsj/.

[36] Sonawane K., Zhu Y., Montealegre J.R. (2020). Parental intent to initiate and complete the human papillomavirus vaccine series in the USA: A nationwide, cross-sectional survey. Lancet Public Health.

[37] Huon J.F., Grégoire A., Meireles A. (2020). Evaluation of the acceptability in France of the vaccine against papillomavirus (HPV) among middle and high school students and their parents. PLoS One.

[38] Karafillakis E., Simas C., Jarrett C. (2019). HPV vaccination in a context of public mistrust and uncertainty: A systematic literature review of determinants of HPV vaccine hesitancy in Europe. Hum Vaccin Immunother.

[39] Lane S., Macdonald N.E., Marti M. (2018). Vaccine hesitancy around the globe: Analysis of three years of WHO/UNICEF Joint Reporting Form data-2015–2017. Vaccine.

[40] Hu H., Wei X., Ren Z. (2014). Investigation on acceptance of HPV vaccination and its determinants among the parents of junior high school students in Guangzhou City. Chin J Dis Control Prev.

[41] Lane S., MacDonald N.E., Marti M., Dumolard L. (2018). Vaccine hesitancy around the globe: Analysis of three years of WHO/UNICEF Joint Reporting Form data-2015-2017. Vaccine.

[42] Wong L.P., Wong P.F., Megat Hashim M.M.A.A. (2020). Multidimensional social and cultural norms influencing HPV vaccine hesitancy in Asia. Hum Vaccin Immunother.

[43] Wang Q., Zhang W., Cai H., Cao Y. (2020). Understanding the perceptions of Chinese women of the commercially available domestic and imported HPV vaccine: A semantic network analysis. Vaccine.

[44] Luisi M.L.R. (2021). From bad to worse II: Risk amplification of the HPV vaccine on Facebook. Vaccine.

[45] Yu Y., Xu M., Sun J. (2016). Human papillomavirus infection and vaccination: Awareness and knowledge of HPV and acceptability of HPV vaccine among mothers of Teenage daughters in Weihai, Shandong, China. PLoS One.

[46] Zhang S.K., Pan X.F., Wang S.M. (2015). Knowledge of human papillomavirus vaccination and related factors among parents of young adolescents: A nationwide survey in China. Ann Epidemiol.

[47] Zhang H., Yu D., Liu C. (2014). Parental acceptance of junior high school students for human papillomavirus vaccination: A survey at one school in Wuhan. J Public Health Prev Med.

[48] Wang W., Ma Y., Wang X. (2015). Acceptability of human papillomavirus vaccine among parents of junior middle school students in Jinan, China. Vaccine.

