# Supplementary material for: Stages of HPV Vaccine Hesitancy Among Guardians of Female Secondary School Students in China
Source: J Adolesc Health. 2023 Jan;72(1):73–9. doi: 10.1016/j.jadohealth.2022.08.027 (PMC9746349; doi:10.1016/j.jadohealth.2022.08.027)
Supplement: Appendix — A [file mmc1.docx]

[Supplemental Material]

Stages of HPV vaccine hesitancy among guardians of female secondary school students in China

Zheng Wei MA.Sc^a,1^, Yang Liu PhD ^b,c,1^, Liuren Zhang MPH ^a1^, Xiu Sun MPH ^a^, Qijing Jiang BA^a^, Zhenwei Li MS^a^, Yue Wu PhD ^a,^ Chuanxi Fu PhD ^a,∗^

^a^ *Institute of Infectious Disease and Vaccine, School of Public Health, Zhejiang Chinese Medical University, No. 548, Binwen Rd, Hangzhou China 310053*

^b^ *Department of Infectious Disease Epidemiology, Faculty of Epidemiology and Population Health, London School of Hygiene and Tropical Medicine, Keppel Street, Bloomsbury, London, England WC1E 7HT*

^c^ *Centre for Mathematical Modelling of Infectious Diseases, London School of Hygiene and Tropical Medicine, Keppel Street, Bloomsbury, London, England WC1E 7HT*

∗ Corresponding author.

*E-mail address:* fuchuanxi@zcmu.edu.cn (C. Fu).

^1^ The authors contributed equally to the article.

Contents

[Supplemental Tables 3](#_Toc87350499)

[Supplemental Table 1： 3](#_Toc87350500)

[Independent t-test of item scores between different PAPM stages 3](#_Toc87350501)

[Supplemental Table 2: 5](#_Toc87350502)

[EFA loadings and CFA standardized regression weights of the HPV Vaccine Hesitancy Scale and the HPV Vaccine Conspiracy Beliefs Scale 5](#_Toc87350503)

[Supplemental Table 3: 7](#_Toc87350504)

[CFA model fits for the scales 7](#_Toc87350505)

[Supplemental Table 4: 8](#_Toc87350506)

[Independent t-test, ANOVA analysis and post-hoc test of scale scores among demographics 8](#_Toc87350507)

[Supplemental Table 5: 9](#_Toc87350508)

[Relationship between HPV related knowledge and PAPM stages 9](#_Toc87350509)

[Supplemental Table 6: 10](#_Toc87350510)

[Self-reported HPV vaccine hesitancy social factors 10](#_Toc87350511)

[Supplemental Table 7: 11](#_Toc87350512)

[Barriers to HPV vaccination 11](#_Toc87350513)

[Supplemental Table 8: 12](#_Toc87350514)

[HPV vaccine preference and free-vaccine acceptance 12](#_Toc87350515)

[Supplemental Methods: 13](#_Toc87350516)

[Questionnaire of HPV vaccine hesitancy for guardians of adolescent aged students 13](#_Toc87350517)

[PART1: Socioeconomic and demographic factors 13](#_Toc87350518)

[PART2: Vaccine hesitancy status 14](#_Toc87350519)

[PART3: HPV vaccine hesitancy scale 15](#_Toc87350520)

[PART4: HPV vaccine conspiracy beliefs scale 15](#_Toc87350521)

[PART5: Social Processes 16](#_Toc87350522)

[PART6: Practical Issues 17](#_Toc87350523)

# Supplemental Tables

## Supplemental Table 1：

### Independent t-test of item scores between different PAPM stages

| **Dimensions** | **HPV Vaccine Hesitancy Scale items** | **Mean score** | |
| --- | --- | --- | --- |
|  |  | **Hesitancy**  **n=1,744** | **No Hesitancy**  **n=1,481** |
| Necessity | The current treatment methods are perfectly advance, and HPV vaccination is not so necessary | 3.53 | 4.09 |
|  | HPV infection can be healed by self-immunity and does not require vaccination | 3.61 | 4.12 |
|  | The government does not mandate HPV vaccination, so it is not necessary | 3.30 | 4.04 |
|  | My daughter is in good health and does not need to be vaccinated against HPV | 3.27 | 4.05 |
|  | My daughter is still in middle school and it is too early to get HPV vaccine | 2.91 | 3.74 |
| Importance | HPV vaccination is the best way to protect my daughter from cervical cancer | 3.61 | 4.19 |
|  | Vaccinating my daughter with HPV can protect her family from infection | 3.52 | 3.94 |
|  | HPV vaccine can effectively prevent cervical cancer | 3.72 | 4.23 |
|  | HPV vaccine can effectively prevent cervical cancer | 3.69 | 4.43 |
|  | HPV vaccination is very important to my daughter’s health | 3.26 | 3.61 |
| Safety | HPV vaccine can effectively prevent some other diseases (such as: condyloma acuminatum) | 2.32 | 2.81 |
|  | I am worried about my daughter having the side effects of HPV vaccine | 2.36 | 2.64 |
|  | I am worried that the HPV vaccine my daughter received is fake or expired | 2.57 | 2.91 |
|  | I am worried about the inappropriate steps the healthcare personnel administering the HPV vaccine | 2.59 | 2.95 |

WHO propose the “3 Cs” model to explain the three categories of vaccine hesitancy (Ref.12,13):

**complacency,** which describes individuals who do not perceive a need for a vaccine (necessity), do not value the vaccine (importance); **convenience,** defined as access to vaccines; **confidence,** defined as trust in the effectiveness and (safety) of vaccines, the system that delivers them, including the reliability and competence of the health services and health professionals and the motivations of policy-makers who decide on the needed vaccines.

In the article, as convenience is more of a behavioral indicator than an attitude indicator, we didn’t include it in the vaccine hesitancy scale. After EFA, three dimensions were extracted, we found that they correspond to: "necessity" and "importance" in complacency, and "safety" in confidence.

| **Dimensions** | **HPV Vaccine Conspiracy Beliefs Scale items** | **Mean score** | |
| --- | --- | --- | --- |
|  |  | **Hesitancy**  **n=1,744** | **No Hesitancy**  **n=1,481** |
|  | Relevant departments or companies concealed adverse events of the HPV vaccine | 3.04 | 2.72 |
|  | Vaccine companies use "hunger marketing" to make HPV vaccine in short supply | 3.07 | 2.80 |
|  | Vaccine companies cut corners when manufacturing HPV vaccines to obtain high profits | 2.79 | 2.49 |
|  | Vaccine company deliberately inflated the price of HPV vaccine | 3.03 | 2.72 |
|  | Vaccine companies work with doctors and persuade people to vaccinate under doctors' advice | 2.91 | 2.52 |
|  | Victims who suffered side effects from the HPV vaccine did not receive reasonable compensation and explanations | 3.03 | 2.73 |
|  | Vaccine company exaggerates the effect of HPV vaccine | 3.02 | 2.58 |
|  | The media and the Internet over-promote the safety and efficacy of HPV vaccination | 3.10 | 2.75 |

Note: The difference between “Hesitancy” and “No Hesitancy” stage for all the scale items is statistically significant(p<0.001).

## Supplemental Table 2:

### EFA loadings and CFA standardized regression weights of the HPV Vaccine Hesitancy Scale and the HPV Vaccine Conspiracy Beliefs Scale

| **Dimensions** | **HPV Vaccine Hesitancy Scale items** | **EFA loadings**  **(n = 699)** | **CFA Standardized regression weights**  **(n =2526)** |
| --- | --- | --- | --- |
| Necessity | The current treatment methods are perfectly advance, and HPV vaccination is not so necessary | 0.855 | 0.732 |
|  | HPV infection can be healed by self-immunity and does not require vaccination | 0.826 | 0.666 |
|  | The government does not mandate HPV vaccination, so it is not necessary | 0.824 | 0.784 |
|  | My daughter is in good health and does not need to be vaccinated against HPV | 0.809 | 0.752 |
|  | My daughter is still in middle school and it is too early to get HPV vaccine | 0.801 | 0.649 |
| Importance | HPV vaccination is the best way to protect my daughter from cervical cancer | 0.787 | 0.723 |
|  | Vaccinating my daughter with HPV can protect her family from infection | 0.779 | 0.533 |
|  | HPV vaccine can effectively prevent cervical cancer | 0.753 | 0.642 |
|  | HPV vaccination is very important to my daughter’s health | 0.714 | 0.703 |
|  | HPV vaccine can effectively prevent some other diseases (such as: condyloma acuminatum) | 0.655 | 0.386 |
| Safety | I am worried about my daughter having the side effects of HPV vaccine | 0.802 | 0.705 |
|  | I am worried that the HPV vaccine my daughter received is fake or expired | 0.836 | 0.736 |
|  | I am worried about the inappropriate steps the healthcare personnel administering the HPV vaccine | 0.814 | 0.711 |
|  | HPV vaccine as a relatively new vaccine still has certain risks | 0.622 | 0.525 |
| **Dimensions** | **HPV Vaccine Conspiracy Beliefs Scale items** |  |  |
|  | Relevant departments or companies concealed adverse events of the HPV vaccine | 0.867 | 0.713 |
|  | Vaccine companies use "hunger marketing" to make HPV vaccine in short supply | 0.841 | 0.720 |
|  | Vaccine companies cut corners when manufacturing HPV vaccines to obtain high profits | 0.921 | 0.735 |
|  | Vaccine company deliberately inflated the price of HPV vaccine | 0.920 | 0.757 |
|  | Vaccine companies work with doctors and persuade people to vaccinate under doctors' advice | 0.913 | 0.723 |
|  | Victims who suffered side effects from the HPV vaccine did not receive reasonable compensation and explanations | 0.849 | 0.686 |
|  | Vaccine company exaggerates the effect of HPV vaccine | 0.899 | 0.710 |
|  | The media and the Internet over-promote the safety and efficacy of HPV vaccination | 0.792 | 0.634 |

Note: EFA = Exploratory Factor Analysis. CFA = Confirmatory Factor Analysis. In EFA, the KMO measure of sampling adequacy was 0.836, and Bartlett’s test of sphericity (p < 0.001) indicated that sufficient correlations among the variables existed. Cronbach’s α for dimensions “Necessity”, “Importance” , “Safety” and “HPV Vaccine Conspiracy Beliefs Scale” was 0.876, 0.821, 0.786 and 0.957 respectively.

## Supplemental Table 3:

### CFA model fits for the scales

|  | **χ^2^ /df** | **SRMR** | **RMSEA** | **CFI** | **TLI** |
| --- | --- | --- | --- | --- | --- |
| HPV Vaccine Hesitancy Scale | 12.927 | 0.0525 | 0.069 | 0.921 | 0.903 |
| HPV Vaccine Conspiracy Beliefs Scale | 17.269 | 0.0323 | 0.080 | 0.964 | 0.949 |
| Suggested value for good fit | <5 | <0.08 | <0.08 | >0.9 | >0.9 |

Note: CFA indices selected to report the model fit are: relative/normed chi-square (χ2 /df); the standardized root mean square residual (SRMR); the root mean square error approximation (RMSEA); the comparative fit index (CFI); the Tucker-Lewis index (TLI). The cutoff criteria were based on “Measuring Model Fit (http://davidakenny.net/cm/fit.htm”).

## Supplemental Table 4:

### Independent t-test, ANOVA analysis and post-hoc test of scale scores among demographics

|  | **Mean(IQR) score** | | | |
| --- | --- | --- | --- | --- |
| **Demographics (n=3,225)** | **Necessity** | **Importance** | **Safety** | **Vaccine Conspiracy Beliefs** |
| Parents gender |  |  |  |  |
| Female (ref.) | 3.71(1.00) | 3.82(0.80) | 2.67(1.00) | 2.77(0.75) |
| Male | 3.47(1.20)*** | 3.75(1.00)** | 2.54(1.00)*** | 3.02(0.75)*** |
| Parents residence |  |  |  |  |
| Urban(ref.) | 3.70(1.00) | 3.86(1.00) | 2.63(1.00) | 2.83(0.88) |
| Rural | 3.48(1.00)*** | 3.63(1.00)*** | 2.62(1.00) | 2.88(0.63) |
| Parents’ education |  |  |  |  |
| Middle school and below (ref.) | 3.45(1.00) | 3.59(1.00) | 2.64(0.75) | 2.89(0.38) |
| Bachelor | 3.69(1.00)*** | 3.86(1.00)*** | 2.63(1.00) | 2.82(0.88) |
| Master and above | 3.77(1.20)*** | 3.89(1.00)*** | 2.58(1.00) | 2.82(0.88) |
| Annual household income |  |  |  |  |
| Decline to answer |  |  |  |  |
| < 7.5 thousand (ref.) | 3.39(1.00) | 3.56(1.00) | 2.59(1.00) | 2.93(0.63) |
| 7.5- thousand | 3.55(1.00)*** | 3.76(0.80)*** | 2.48(1.00) | 3.01(0.75) |
| 15- thousand | 3.69(1.00)*** | 3.86(1.00)*** | 2.62(1.00) | 2.83(0.88) |
| 30- thousand | 3.88(1.00)*** | 3.97(0.80)*** | 2.76(1.25)** | 2.66(1.00)*** |
| 45- thousand | 3.86(1.00)*** | 4.01(0.80)*** | 2.77(1.50)** | 2.71(1.00)** |
| Daughter age |  |  |  |  |
| Middle school student(age 12-15) (ref.) | 3.66(1.20) | 3.81(0.80) | 2.64(1.00) | 2.83(0.88) |
| High school students / technical school students / vocational school students(age 16-19) | 3.59(1.20)* | 3.77(1.00) | 2.61(1.00) | 2.88(0.75)* |

Note: Dunnett-t test was used in post-hoc test.

“*” means p-value<0.05; “**” means p-value<0.01; “***” means p-value<0.001.

## Supplemental Table 5:

### Relationship between HPV related knowledge and PAPM stages

| **HPV related-knowledge** | **Percent (%)** | **PAPM stages** | | |
| --- | --- | --- | --- | --- |
|  |  | **Hesitancy**  **n=1,744** | **No Hesitancy**  **n=1,481** | **OR(95%CI)** |
| HPV infection can be spread through sexual activities |  |  |  |  |
| Don’t Know | 40.4 | 840 (48.2%) | 402 (27.1%) | 2.05 (1.77-2.37) *** |
| Know | 59.6 | 904 (51.8%) | 1019 (72.9%) |  |
| HPV vaccine is best recommened before the first sexual activities |  |  |  |  |
| Don’t Know | 45.3 | 997 (57.2%) | 461 (31.1%) | 2.95 (2.55-3.42) *** |
| Know | 54.7 | 747 (42.8%) | 1020 (68.9%) |  |
| HPV vaccine usually requires 2-3 doses |  |  |  |  |
| Don’t Know | 59.1 | 1241 (71.2%) | 668 (45.1%) | 3.00 (2.60-3.47) *** |
| Know | 40.9 | 503 (28.8%) | 813 (54.9%) |  |
| The protective effect of HPV vaccine usually lasts for more than 10 years |  |  |  |  |
| Don’t Know | 72.5 | 1428 (81.9%) | 908 (61.3%) | 2.85 (2.43-3.35) *** |
| Know | 27.5 | 316 (18.1%) | 573 (38.7%) |  |

Note: Chi-square analysis was used to calculate OR, “Hesitancy” stage was set as control.

“***” means p-value<0.001.

## Supplemental Table 6:

### Self-reported HPV vaccine hesitancy social factors

| **HPV vaccine hesitancy social factors** | **Percent (%)** | **PAPM stages** | | |
| --- | --- | --- | --- | --- |
|  |  | **Hesitancy**  **n=1,744** | **No Hesitancy**  **n=1,481** | **OR(95%CI)** |
| Public health doctors |  |  |  |  |
| Yes | 43.8 | 657 (37.7%) | 755 (51.0%) | 1.72(1.49-1.98)  *** |
| No | 56.2 | 1087 (62.3%) | 726 (49.0%) |  |
| Clinical doctors |  |  |  |  |
| Yes | 58.3 | 888 (50.9%) | 992 (67.0%) | 1.95(1.69-2.25)  *** |
| No | 41.7 | 856 (49.1%) | 489 (33.0%) |  |
| Colleagues/Friends/Neighbors |  |  |  |  |
| Yes | 38.1 | 602 (34.5%) | 626 (42.3%) | 1.40(1.21-1.61)  *** |
| No | 61.9 | 1142 (65.5%) | 855 (57.7%) |  |
| Family members |  |  |  |  |
| Yes | 42.1 | 677 (38.8%) | 681 (46.0%) | 1.34(1.17-1.54)  *** |
| No | 57.9 | 1067 (61.2%) | 800 (54.0%) |  |
| Social media |  |  |  |  |
| Yes | 42.8 | 645 (37.0%) | 735 (49.6%) | 1.68(1.46-1.93)  *** |
| No | 57.2 | 1099 (63.0%) | 746 (50.4%) |  |
| Public figure |  |  |  |  |
| Yes | 38.1 | 584 (33.5%) | 644 (43.5%) | 1.54(1.33-1.77)  *** |
| No | 61.9 | 1160 (66.5%) | 837 (56.5%) |  |

Note: Chi-square analysis was used to calculate OR, “Hesitancy” stage was set as control.

“***” means p-value<0.001.

## Supplemental Table 7:

### Barriers to HPV vaccination

| **HPV related-knowledge** | **Percent (%)** | **PAPM stages** | | |
| --- | --- | --- | --- | --- |
|  |  | **Decided**  **n=1,239** | **Vaccinated**  **n=242** | **OR(95%CI)** |
| Do not know where to go to receive HPV vaccine |  |  |  |  |
| Barrier | 46.8 | 591 (47.7%) | 103 (42.6%) | 1.23 (0.93-1.63) |
| No Barrier | 53.2 | 648 (52.3%) | 139 (57.4%) |  |
| Not enough time to get HPV vaccination due to busy schedule |  |  |  |  |
| Barrier | 36.4 | 466 (37.6%) | 74 (30.6%) | 1.37 (1.02-1.84) * |
| No Barrier | 63.6 | 773 (62.4%) | 168 (69.4%) |  |
| It is difficult to make an HPV vaccine appointment |  |  |  |  |
| Barrier | 77.0 | 981 (79.2%) | 160 (66.1%) | 1.95 (1.44-2.63) *** |
| No Barrier | 23.0 | 258 (20.8%) | 82 (33.9%) |  |
| Personal thought: the price of the HPV vaccine is too high |  |  |  |  |
| Barrier | 62.1 | 769 (62.1%) | 151 (62.4%) | 0.99 (0.72-1.31) |
| No Barrier | 37.9 | 470 (37.9%) | 91 (37.6%) |  |

Note: Chi-square analysis was used to calculate OR, “Decided” stage was set as control.

“*” means p-value<0.05; “***” means p-value<0.001.

## Supplemental Table 8:

### HPV vaccine preference and free-vaccine acceptance

| **PAPM stages** | **Which kind of HPV vaccine do you prefer?** | | | | | | | | |
| --- | --- | --- | --- | --- | --- | --- | --- | --- | --- |
|  | **Don't know** | **2-valent** | **4-valent** | | **9-valent** | **2,4-valent** | | **4,9-valent** | **2,4,9-valent** |
| Vaccinated (n=242) | 35.5% | 9.6% | 16.5% | | 28.5% | 2.2% | | 2.4% | 5.4% |
| Decided (n=1239) | 33.4% | 7.9% | 10.8% | | 36.3% | 2.4% | | 7.1% | 2.1% |
| Totally (n=1481) | 33.7% | 8.1% | 11.7% | | 34.9% | 2.3% | | 6.8% | 2.5% |
|  | **Would you like to be vaccinated if HPV vaccine was provided for free?** | | | | | | | | |
|  | **Yes** | | | **Still need to consider** | | | **No** | | |
| Unaware (n=460) | 55.4% | | | 40.9% | | | 3.7% | | |
| Unengaged (n=572) | 66.6% | | | 28.0% | | | 5.4% | | |
| Refused (n=31) | 41.9% | | | 38.7% | | | 19.4% | | |
| Undecided (n=681) | 80.3% | | | 18.4% | | | 1.3% | | |
| Totally (n=1744) | 68.6% | | | 27.8% | | | 3.6% | | |

# Supplemental Methods:

## Questionnaire of HPV vaccine hesitancy for adolescent aged female students guardians

### PART1: Socioeconomic and demographic factors

1. Do you have a daughter who is in junior high school or high school ?

| ○Yes |
| --- |
| ○No (END) |

2. Gender

| ○female |
| --- |
| ○male |

3. Residence

| ○urban |
| --- |
| ○rural |

4. Education

| ○Middle school and below |
| --- |
| ○Junior college |
| ○College |
| ○Postgraduate and above |

5. Income(USD)

| ○< 7.5 thousand |
| --- |
| ○7.5-15 thousand |
| ○15-30 thousand |
| ○30-45 thousand |
| ○≥ 45 thousand |
| ○Decline to answer |

6. Daughter age

○Middle school student(age 12-15)

○High school students / technical school students / vocational school students(age 16-19)

### PART2: Vaccine hesitancy status

7. Regarding your daughter's HPV vaccination, which of the following is most suitable for your current state?

| ○“Unaware”: Never heard of HPV vaccine (jump to item 9) |
| --- |
| ○“Unengaged”: Know about the existence of HPV vaccine, but never considered getting HPV vaccine (jump to item 9) |
| ○“Undecided”: Considered before but not yet decided on administering HPV vaccine(jump to item 9) |
| ○“Refuse”: Refuse to administer HPV vaccine (jump to item 9) |
| ○“Decided”: Decided to take HPV vaccine but not yet vaccinated due to practical barriers  ○“Vaccinated”: Administering/Vaccinated with HPV vaccine |

8. Which kind of HPV vaccine do you prefer? (jump to item 10)

| ○2-valent |
| --- |
| ○4-valent |
| ○9-valent |
| ○Don't know the difference   1. Would you like to be vaccinated if HPV vaccine was provided for free? |

○Yes

○Still need to consider

○No

### PART3: HPV vaccine hesitancy scale

| **HPV Vaccine Hesitancy Scale items** | **5** | **4** | **3** | **2** | **1** |
| --- | --- | --- | --- | --- | --- |
| 10.The current treatment methods are perfectly advance, and HPV vaccination is not so necessary |  |  |  |  |  |
| 11.HPV infection can be healed by self-immunity and does not require vaccination |  |  |  |  |  |
| 12.The government does not mandate HPV vaccination, so it is not necessary |  |  |  |  |  |
| 13.My daughter is in good health and does not need to be vaccinated against HPV |  |  |  |  |  |
| 14.My daughter is still in middle school and it is too early to get HPV vaccine |  |  |  |  |  |
| 15.Young girls vaccinated against HPV may be perceived by others to be more sexually active |  |  |  |  |  |
| 16.HPV vaccination is the best way to protect my daughter from cervical cancer |  |  |  |  |  |
| 17.Vaccinating my daughter with HPV can protect her family from infection |  |  |  |  |  |
| 18.HPV vaccine can effectively prevent cervical cancer |  |  |  |  |  |
| 19.HPV vaccine can effectively prevent cervical cancer |  |  |  |  |  |
| 20.HPV vaccination is very important to my daughter’s health |  |  |  |  |  |
| 21.HPV vaccine can effectively prevent some other diseases (such as: condyloma acuminatum) |  |  |  |  |  |
| 22.I am worried about my daughter having the side effects of HPV vaccine |  |  |  |  |  |
| 23.I am worried that the HPV vaccine my daughter received is fake or expired |  |  |  |  |  |
| 24.I am worried about the inappropriate steps the healthcare personnel administering the HPV vaccine |  |  |  |  |  |

### PART4: HPV vaccine conspiracy beliefs scale

| **HPV Vaccine Conspiracy Beliefs Scale items** | **5** | **4** | **3** | **2** | **1** |
| --- | --- | --- | --- | --- | --- |
| 25.Relevant departments or companies concealed adverse events of the HPV vaccine |  |  |  |  |  |
| 26.Vaccine companies use "hunger marketing" to make HPV vaccine in short supply |  |  |  |  |  |
| 27.Vaccine companies cut corners when manufacturing HPV vaccines to obtain high profits |  |  |  |  |  |
| 28.Vaccine company deliberately inflated the price of HPV vaccine |  |  |  |  |  |
| 29.Vaccine companies work with doctors and persuade people to vaccinate under doctors' advice |  |  |  |  |  |
| 30.Victims who suffered side effects from the HPV vaccine did not receive reasonable compensation and explanations |  |  |  |  |  |
| 31.Vaccine company exaggerates the effect of HPV vaccine |  |  |  |  |  |
| 32.The media and the Internet over-promote the safety and efficacy of HPV vaccination |  |  |  |  |  |

### PART5: Social Processes

1. HPV infection can be spread through sexual activities

○Don’t Know

○Know

1. HPV vaccine is best recommened before the first sexual activities

○Don’t Know

○Know

1. HPV vaccine usually requires 2-3 doses

○Don’t Know

○Know

1. The protective effect of HPV vaccine usually lasts for more than 10 years

○Don’t Know

○Know

1. The recommendations of public health doctors on the HPV vaccine have a great impact on me

○Yes

○No

1. The recommendations of clinical doctors on the HPV vaccine have a great impact on me

○Yes

○No

1. The recommendations of colleagues/friends/neighbors on the HPV vaccine have a great impact on me

○Yes

○No

1. The recommendations of family members on the HPV vaccine have a great impact on me

○Yes

○No

1. The recommendations of social media on the HPV vaccine have a great impact on me

○Yes

○No

1. The recommendations of public figure on the HPV vaccine have a great impact on me

○Yes

○No

### PART6: Practical Issues

1. Do not know where to go to receive HPV vaccine

○Barrier

○No Barrier

1. Not enough time to get HPV vaccination due to busy schedule

○Barrier

○No Barrier

1. It is difficult to make an HPV vaccine appointment

○Barrier

○No Barrier

1. Personal thought: the price of the HPV vaccine is too high

○Barrier

○No Barrier
